# Neuronal Differentiation and Outgrowth Effect of Thymol in *Trachyspermum ammi* Seed Extract via BDNF/TrkB Signaling Pathway in Prenatal Maternal Supplementation and Primary Hippocampal Culture

**DOI:** 10.3390/ijms24108565

**Published:** 2023-05-10

**Authors:** Binod Timalsina, Md Nazmul Haque, Raju Dash, Ho Jin Choi, Nisha Ghimire, Il Soo Moon

**Affiliations:** 1 Department of Anatomy, Dongguk University College of Medicine, Gyeongju 38066, Republic of Korea; binodtimalsina19@gmail.com (B.T.); habib.uni.ac.bd@gmail.com (M.N.H.); rajudash.bgctub@gmail.com (R.D.); chjack@naver.com (H.J.C.); 2 Department of Fisheries Biology and Genetics, Patuakhali Science and Technology University, Patuakhali 8602, Bangladesh; 3 Department of Life Science and Biochemical Engineering, Graduate School, Sun Moon University, Asan 31460, Republic of Korea; nysaghimire8@gmail.com

**Keywords:** BDNF, hippocampal neurons, neuritogenesis, MAP2, thymol, *Trachyspermum ammi*

## Abstract

Reviving the neuronal functions in neurodegenerative disorders requires the promotion of neurite outgrowth. Thymol, which is a principal component of *Trachyspermum ammi* seed extract (TASE), is reported to have neuroprotective effects. However, the effects of thymol and TASE on neuronal differentiation and outgrowth are yet to be studied. This study is the first report investigating the neuronal growth and maturation effects of TASE and thymol. Pregnant mice were orally supplemented with TASE (250 and 500 mg/kg), thymol (50 and 100 mg/kg), vehicle, and positive controls. The supplementation significantly upregulated the expression of brain-derived neurotrophic factor (BDNF) and early neuritogenesis markers in the pups’ brains at post-natal day 1 (P1). Similarly, the BDNF level was significantly upregulated in the P12 pups’ brains. Furthermore, TASE (75 and 100 µg/mL) and thymol (10 and 20 µM) enhanced the neuronal polarity, early neurite arborization, and maturation of hippocampal neurons in a dose-dependent manner in primary hippocampal cultures. The stimulatory activities of TASE and thymol on neurite extension involved TrkB signaling, as evidenced by attenuation via ANA-12 (5 µM), which is a specific TrkB inhibitor. Moreover, TASE and thymol rescued the nocodazole-induced blunted neurite extension in primary hippocampal cultures, suggesting their role as a potent microtubule stabilizing agent. These findings demonstrate the potent capacities of TASE and thymol in promoting neuronal development and reconstruction of neuronal circuitry, which are often compromised in neurodegenerative diseases and acute brain injuries.

## 1. Introduction

Neurodegenerative and psychiatric disorders are the outcomes of alterations in several factors; the neurotrophic factors are one of the leading causes of neuronal death, emphasizing their critical roles in regulating neuronal functions [1]. Neurotrophic factors comprise essential secreted proteins having crucial roles in the cellular and molecular mechanisms determining cell survival, maintenance, and excitability in the peripheral and central nervous system (CNS) [2,3].

Neurotrophins are a family of proteins that maintain neuronal survival, neurite expansion, and influence neuronal and glial cell differentiation during development and injury [4]. Regulation of neurotrophins, such as brain-derived neurotrophic factor (BDNF) and nerve growth factor (NGF), plays a significant role in the maintenance and differentiation of the developing and mature neurons [5]. In early neuronal development, BDNF and its receptor tropomyosin-related kinase receptor type B (TrkB) signaling regulate phosphorylation and local translation of downstream proteins, promoting dendritic and axonal development via modulating actin and microtubule dynamics [6]. In addition to the BDNF, several regulatory proteins play important roles in early neuronal differentiation and neurite outgrowth activity. For example, neuritin 1 (NRN1), which is a signaling molecule downstream of BDNF/TrkB, facilitates neuritogenesis in primary hippocampal and cortical cultures [7]. As another crucial cytoskeletal modulator, as well as a neuronal marker, the microtubule-associated protein 2 (MAP2) functions in the nucleation and stabilization of microtubules, regulates microtubule-dependent transport in axons and dendrites, and provides anchorage for regulatory proteins, such as kinases, phosphatases, and neurotransmitter receptors [8,9]. Moreover, N-acetylglucosamine kinase (NAGK) was reported from our laboratory to promote neuronal arborization, suggesting its crucial neurotrophic role [10,11].

The development of agents that sustain BDNF/TrkB signaling and other associated proteins would be one therapeutic target in neurodegenerative disorders. Natural products are a reservoir for such agents; studied extensively, they demonstrate neurotrophic activity via enhancing axonal and dendritic maturation and synaptogenesis [12,13,14]. As a continuous effort to explore potent and druggable natural neuritogenic factor mimetic therapeutic agents, this study is aimed at observing the neuronal differentiation and maturation potential of *Trachyspermum ammi* seed extract (TASE) and its principal component, known as thymol, in vivo through pre-natal maternal supplementation and in rat primary hippocampal neurons in vitro. Mechanistic insights into the TASE and thymol supplementation effect in a neuron are also discerned. *T. ammi* is a traditional aromatic herb harboring several therapeutic potentials against diverse diseases, such as antioxidant, anti-inflammatory, antinociceptive, antimicrobial, antitumor, and neuroprotective activities [15,16,17]. Although neuroprotective effects of TASE are abundant, neurite outgrowth potential is not reported. Considering these traditional benefits of TASE, this study aimed to disclose the neuritogenic potential of TASE and its principle component, known as thymol, in pre-natal maternal supplementation and primary hippocampal neurons.

In this study, we investigated the effects of TASE and thymol in the early neuronal differentiation and maturation in pre-natal maternal supplementation and primary hippocampal cultures. The results show that TASE and thymol promote neuronal differentiation, while axodendritic outgrowth upregulates the BDNF/TrkB signaling, along with its associated early neuritogenesis markers.

## 2. Results

### 2.1. Identification of Thymol in TASE

*T. ammi* seed extract (TASE) was prepared with 95% ethanol at room temperature. Ultra-high performance liquid chromatography (U-HPLC) was performed to identify the phenolic compounds present in the extract (Figure S1). Since thymol is the major component in TASE, we used the standard compound thymol to compare the peaks in the extract [18]. Thymol was observed at the retention time of 17.106 min, which exactly matched that of the standard peak, hence confirming the principal component in TASE. The amount of thymol present in the TASE extract was 33.7%, quantified via the total peak area in U-HPLC, as reported previously [17]. Further experiments were conducted with TASE and thymol for the neuritogenesis and maturation of hippocampal neurons in rat primary culture and in vivo pre-natal supplementation.

### 2.2. TASE and Thymol Treatment in the Pregnant Mice Upregulated the Neurite Growth Factors in the Pups In Vivo

**Figure 1:**
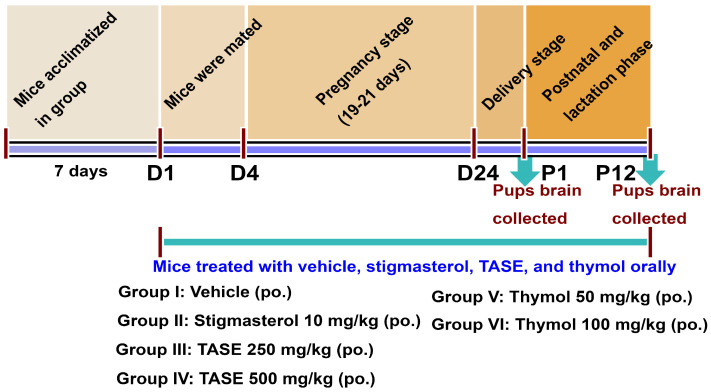
Scheme showing the overall in vivo experimental plan. The female mice 3 months old (*n* = 4 in each group) were grouped and acclimatized for a week. The treatment was started with vehicle, stigmasterol, TASE, and thymol orally with a single dose per day. Mice were mated after starting the treatment from days D1 to D4 and males were separated. Treatment was continuous throughout the pregnancy and lactation stage (D1-P12). After the delivery, pups were sacrificed at P1; the brains were collected for further analysis. The mothers’ mice were fed with the TASE and thymol including the controls continuously until the pups were at P12 days and the pups’ brains were collected for analysis.

**Figure 2:**
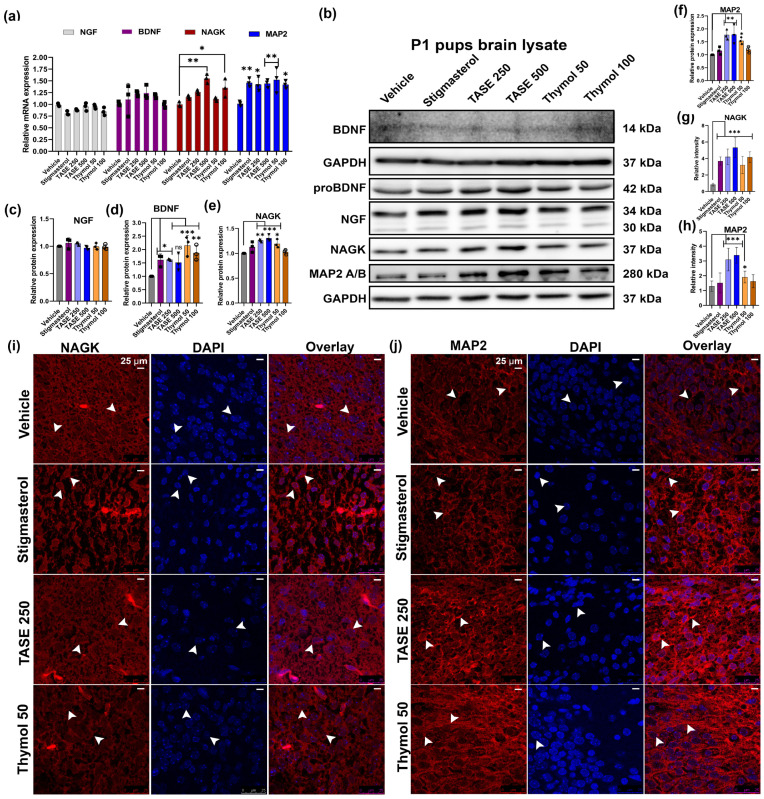
In vivo experiment showing neurite outgrowth effects in mouse pups delivered from TASE-, thymol-, and stigmasterol-treated mothers. (**a**) RT-qPCR analysis of P1 pups’ brain total RNA. (**b**) Western blot analysis of pups’ brain lysates. (**c**–**f**) Relative quantification of western blot intensity. (**g**,**h**) Relative intensity measurement of immuno-histochemistry (IHC) staining for NAGK and MAP2 expression in P1 pups brain. (**i**,**j**) IHC staining for NAGK and MAP2 expression in P1 pups’ brains. Scale bar, 25 μm, is applied to all images. Arrowheads represent the cell body and process of the neurons. One- and two-way ANOVAs with Dunnett’s and Tukey’s multiple comparisons were used for statistical analysis. * *p* < 0.05, ** *p* < 0.01, and *** *p* < 0.001 represent difference between vehicle control and treatment groups. (*n* = 3 pups from different mothers in same treatment group).

Female mice were treated with TASE extracts along with the compound thymol, positive control stigmasterol, and vehicle, as shown in Figure 1. After the delivery, pups were sacrificed on the P1 stage, and the collected brain samples were used for further analysis. Total RNA was isolated from the whole brain samples. RT-qPCR was performed on the P1 total RNA from the pups’ brains of different treatment groups. The early neurites outgrowth markers, such as NGF, BDNF, NAGK, MAP2, and NRN1, were observed via RT-qPCR using the primers shown in Table S1. TASE and thymol increased the relative expression of these genes compared to the vehicle control group, with significant differences in NAGK and NRN1 genes, as shown in Figure 2a and supplementary Figure S3. To validate the RT-qPCR data, we performed the western blot analysis (Figure 2b–f) of the pups’ brain lysate at P1, showing the significant differences in the NAGK (*p* < 0.05) and MAP2 (*p* < 0.05) protein expression, which are the early markers of neuritogenesis and neuronal differentiation. In addition, the mature BDNF blot at the P1 stage was blurred due to the minimal concentration of the protein. To further validate the TASE and thymol effect on BDNF upregulation, we analyzed the P12 mouse pups’ brain lysate. The mature BDNF level was significantly upregulated in the TASE 500 mg/kg- (*p* < 0.01) and thymol 50 mg/kg (*p* < 0.001)-treated groups (Figure S4a,b). Accordingly, the BDNF level was also significantly upregulated in the scopolamine-induced Alzheimer’s disease (AD) model of adult mouse brains following the TASE and thymol treatments in our previous report [17]. Western blot data were further validated via immuno-histochemistry and intensity analysis of the protein expression in the brain (Figure 2g–j). NAGK and MAP2 levels were significantly increased (*p* < 0.05) in the TASE- and thymol-treated groups.

### 2.3. TASE and Thymol Promote Neurite Outgrowth in Prenatal Supplementation

**Figure 3:**
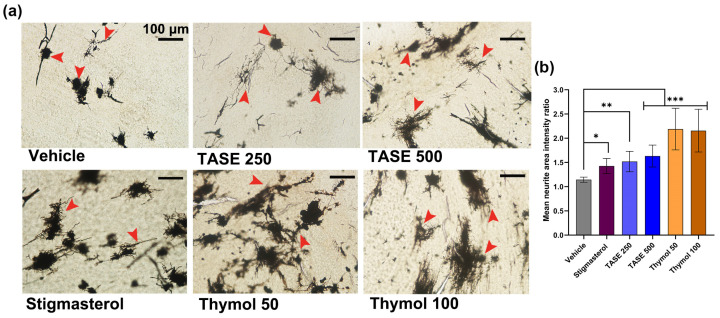
Golgi–Cox staining exhibiting neurite outgrowth effect of TASE and thymol in pre-natal supplementation at P1 pups brain. (**a**) Representative images demonstrating neuronal differentiation and outgrowth effect. Red arrow indicates significant neuron. Here, pre-natal supplementation and groups are same as in Figure 1. Scale bar, 100 μm, is applied to all images. (**b**) Mean neurite area intensity ratio between different treatment groups. Ratio between neurite intensity and background intensity with equal area was averaged, and one-way ANOVA with Dunnett’s multiple comparisons post hoc test was used for comparison between groups. (*n* = 3 pups from different mothers of same treatment group). * *p* < 0.05, ** *p* < 0.01, and *** *p* < 0.001 represent difference between vehicle control and treatment groups.

Next, we performed Golgi–Cox staining of the pups’ brains isolated at post-natal day 1 (P1) from the mothers supplemented with the vehicle, TASE, thymol, and positive control stigmasterol. TASE and thymol exhibited prominent neurite outgrowth (*p* < 0.001) compared with the vehicle control. Specifically, measurements of mean neurite area intensity showed that the supplementation of TASE (250 and 500 mg/kg) and thymol (50 and 100 mg/kg) demonstrated more neuronal complexity than the vehicle group, confirming their neurotrophic potential in the pups’ neurons (Figure 3).

### 2.4. Determination of Optimal Dose for TASE and Thymol for Neurite Outgrowth Activity in Primary Hippocampal Culture

**Figure 4:**
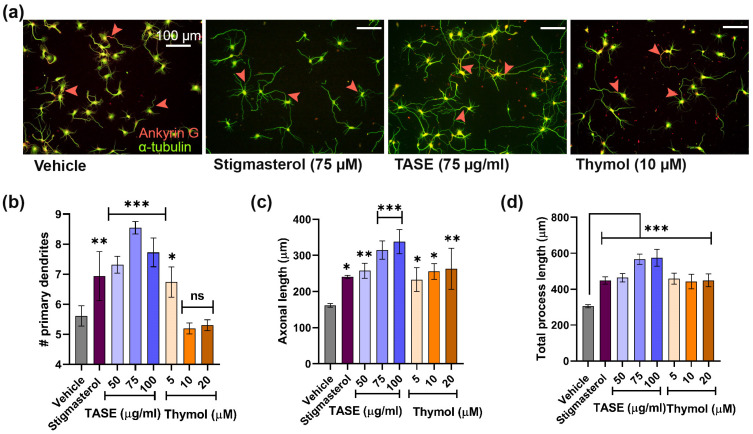
Determination of optimal concentration of TASE and thymol for neurite outgrowth activity. (**a**) Immunofluorescence images for cultures incubated with vehicle (DMSO), positive control (stigmasterol), ethanolic extracts of TASE, and thymol compound with optimal concentration. Whole neuron morphology was stained with alpha-tubulin (green) and axon initial segment marker, known as ankyrin G (red). Arrowhead represents the axon hillock stained with ankyrin G. (**b**) Number of primary processes. (**c**) Length of longest process. (**d**) Total length of primary processes exhibits neurite outgrowth promoting activity of TASE and thymol. A scale bar, 100 μm, is applied to all the images. Statistical significance is compared to vehicle with *p* values: * *p* < 0.05, ** *p* < 0.01, *** *p* < 0.001, and ns: not significant (one–way ANOVA with Dunnett’s multiple comparisons test). Data points are represented as mean ± SD (*n* ≥ 30 individual neurons from three independent experiments). SD, standard deviation.

The neurotrophic factor mimetic activity of TASE and thymol was determined on the E19 rat hippocampal cultures at variable doses. The number of neurons and glia in the culture system were determined through immuno-cytochemistry using a neuron- (MAP2) and glia-specific (GFAP) markers at DIV 3 and DIV 14 (Figure S2a,b). We observed fewer than 5% glial cells at DIV 3 and around 8% glial cells at DIV 14 (Figure S2c). The effective dose was confirmed via treating TASE (50–150 μg/mL) and thymol (5–20 μM) concentrations in the hippocampal culture media as a screening experiment for the first 3 days. The neuritogenesis effect was compared with the known positive control stigmasterol (75 μM) and the vehicle control. Morphometric analysis, such as the number of primary processes (neurites that originated directly from the soma) and the length of the primary process (sum of the length of the primary process), was performed for the TASE- and thymol-treated neuron for neurite outgrowth activity (Figure 4a). TASE exhibited significant maximal activity at 75 μg/mL (*p* < 0.001) for the primary process count, whereas the length of the primary process and the total process were maximal at 100 μg/mL (*p* < 0.001) concentration. Thus, both doses of TASE were studied for further analysis (Figure 4b–d). We also determined the optimal concentration for thymol at 10–20 μM (*p* < 0.05), where it exhibited maximal neurite outgrowth activity (Figure 4b–d). Moreover, we confirmed the cell viability using a trypan blue exclusion assay for the optimal doses of TASE and thymol in hippocampal cultures (Figure S5).

### 2.5. TASE and Its Active Constituent Thymol Promote Neuronal Differentiation

Neurons are polarized cells with one axon and multiple dendrites. Neurons attain this polarity stereotypically in the very early developmental stage. We observed the possible effects of TASE and thymol on neuronal polarity. For this purpose, we categorized neurons into different developmental stages based on their morphology. The neuronal cell populations at different developmental stages were counted using captured images. Specific markers to dendrites (anti-MAP2 antibody labeled) were used to differentiate between the developmental stages.

**Figure 5:**
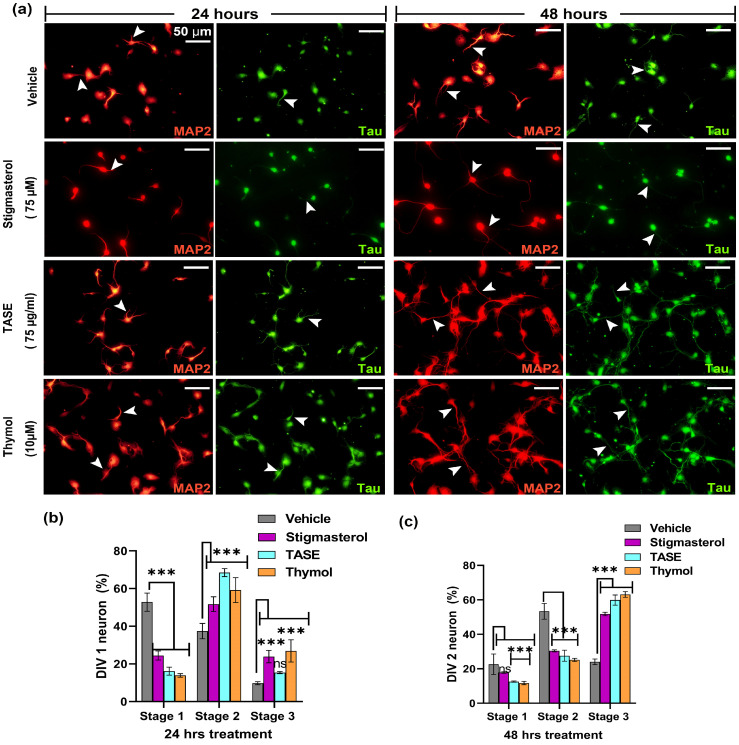
Effects of TASE and thymol on early neuronal differentiation. Hippocampal cultures were incubated with TASE and thymol for 24 h and 48 h with vehicle and stigmasterol (75 µM) as control, with dendrite stained with MAP2 (red) as a dendrite-specific marker and tau (green) as an axon marker. (**a**) Immunofluorescence images showing neuronal differentiation and outgrowth (axonal and dendritic sprouting, indicated with arrowheads). Arrowheads indicate the axonal and dendritic processes of the neuron. Scale bar, 50 μm applies to all images. (**b**) Percentage of neurons that reached different developmental stages at 24 h and (**c**) 48 h of incubation. Statistical significance is compared to vehicle with the *p*-values: *** *p* < 0.001, and ns (not significant) (one- and two-way ANOVAs with Dunnett’s and Tukey’s multiple comparisons tests). Data points are represented as mean ± SD (*n* = 500 individual neurons from three independent experiments). SD, standard deviation.

After 24 h of treatment of TASE and thymol in hippocampal culture, we categorized the stages of polarization as stage 1 (lamellipodia stage), stage 2 (minor process stage), and stage 3 (axonal sprouting) (Figure 5a). We observed a significant variation (*p* < 0.001) in the neuronal polarity in the TASE- and thymol-treated neurons compared with the vehicle control (Figure 5b,c). In 24 h, most of the neurons in the vehicle group were in stage 1, whereas stigmasterol-, TASE-, and thymol-treated neurons were mostly in stages 2 and 3. Specifically, 27% of thymol-treated neurons were in stage 3 (axon development), which is 3-fold more than in the vehicle control (9% of neurons). Similarly, in 48 h, 2/3 of the TASE- and thymol-treated neurons were in stage 3, whereas a similar portion of vehicle-treated neurons was in developmental stage 2 (minor processes). These results indicate the early neuronal differentiation potential of both TASE and thymol.

### 2.6. TASE and Thymol Exert Neurite Outgrowth Activity via the TrkB Signaling Pathway

**Figure 6:**
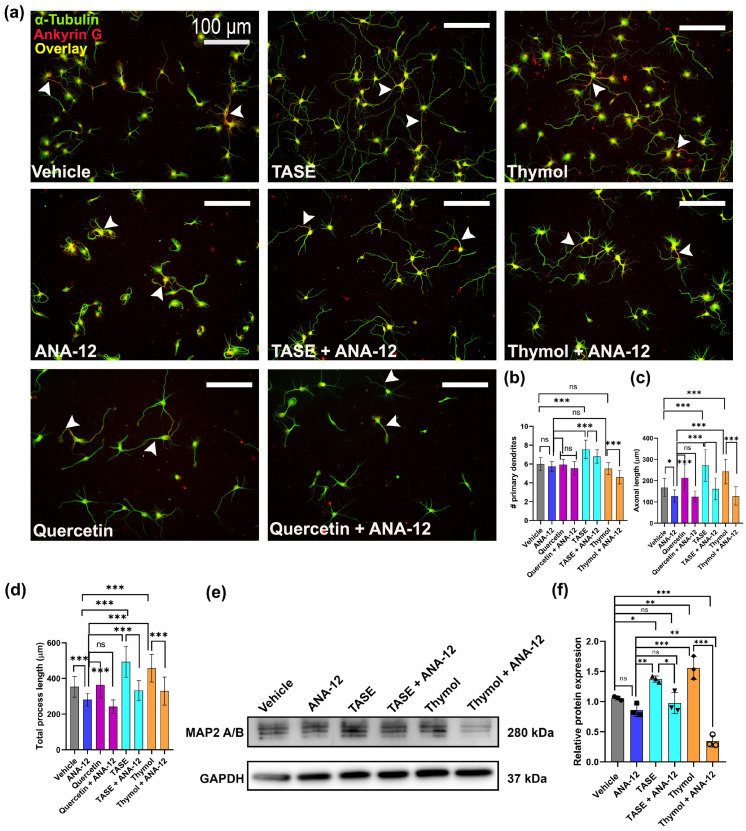
TASE and thymol exert neurite outgrowth activity via TrkB signaling pathway. Primary hippocampal neurons were maintained under same culture conditions, as indicated in Figure 4. For 3 days in presence of vehicle, ANA-12 (TrkB inhibitor, 5 μM), TASE (75 μg/mL) or TASE + ANA-12, thymol (10 μM) or thymol (10 μM) + ANA-12, and quercetin (5 μM) or quercetin (5 μM) + ANA-12 (**a**) Representative immunofluorescence images with α-tubulin (green) and ankyrin G (red) from each treatment. Arrowheads represent the axon hillock stained via ankyrin G (red). Scale bar, 100 μm, applies to all images. (**b**) Number of primary processes, (**c**) length of longest primary process, and (**d**) length of total primary processes. (**e**) Western blot image showing MAP2 protein expression. (**f**) Bar graphs showing relative quantification of western blot intensity. Bars represent mean ± SD (*n* = 30 neurons) and (*n* = 3) for western blot analysis. Statistical significance was compared to vehicle: * *p* < 0.05, ** *p* < 0.01, *** *p* < 0.001, and ns: not significant, one-way ANOVA with Dunnett’s multiple comparisons tests.

Next, we investigated whether TASE and thymol exerted neurotrophic effects through a canonical signaling pathway. Treatment of neuronal culture with a specific TrkB inhibitor (5 μM), known as ANA-12, significantly attenuated the stimulatory activity of TASE and thymol on neurite extension, indicating that TASE- and thymol-mediated neurite outgrowth involves the TrkB signaling pathway (Figure 6a). We used the TrkB agonist compound quercetin (5 μM) as the positive control in the study [19]. The treated neurons were fixed after 3 days and immunostained with α-tubulin (green), and bright-field images were used for morphometric analysis. The number of primary processes was reduced (*p* < 0.001) using the TrkB inhibitor in both the TASE and thymol treatments (Figure 6b). The TrkB inhibitor significantly reduced (*p* < 0.001) the length of the longest process (Figure 6c) and total length of the primary process (Figure 6d) in all the treatments, including the positive control quercetin. Western blot analysis was performed after 3 days of treatment with ANA-12 and treatment alone with controls (vehicle and ANA-12) (Figure 6e). We observed significant upregulation (*p* < 0.001) of the MAP2 protein level in TASE- and thymol-treated groups, whereas a 3-fold reduction in the MAP2 level was observed in the combined ANA-12- and thymol-treated group (Figure 6f). These results indicate that TASE and thymol exert neurotrophic activity through the TrkB-mediated signaling pathway.

### 2.7. TASE and Thymol Can Rescue the Nocodazole-Induced Microtubule Depolymerization

**Figure 7:**
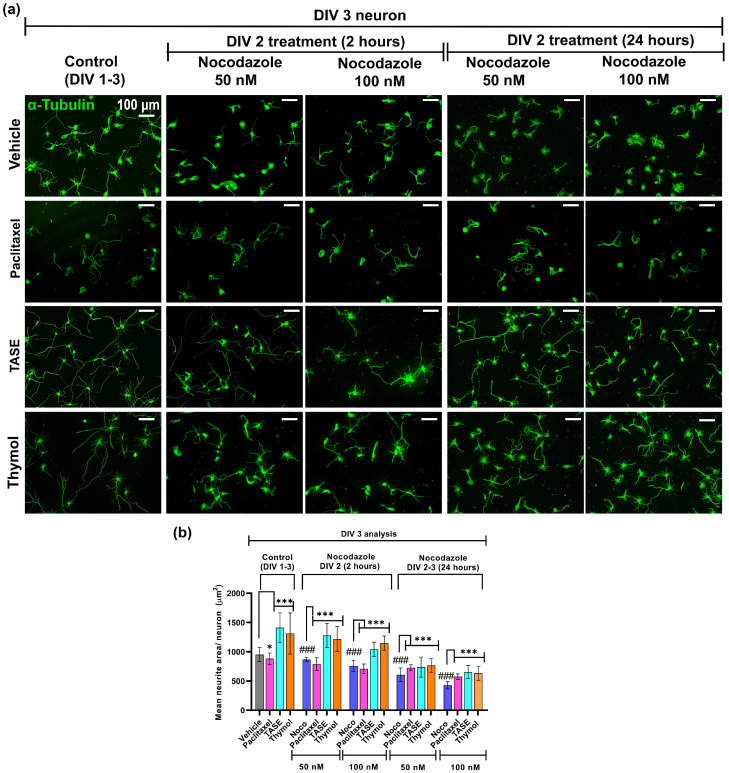
TASE and thymol can rescue nocodazole-induced microtubule depolymerization. Primary hippocampal culture maintained with vehicle, positive control paclitaxel (10 nM), TASE (75 µg/mL), and thymol (10 µM) were treated with nocodazole 50 and 100 nM on DIV 2 for 2 and 24 h, respectively. On DIV 3, neurons were fixed and immunostained with α-tubulin antibody (green). (**a**) Immunostained images with α-tubulin antibody (green) from each treatment in 2 (50 and 100 nM nocodazole) and 24 h (50 and 100 nM nocodazole), respectively. Scale bar, 100 μm, applies to all images. (**b**) Mean neurite area for each treatment. NeurphologyJ plug-in and mean area (µm^2^) per neuron were calculated via dividing total neurites area by total soma count. Bars represent mean ± SD (*n* > 150 neurons)**.** Statistical significance was compared to vehicle, vehicle, and nocodazole (### *p* < 0.001): * *p* < 0.05, and *** *p*< 0.001 (one- and two-way ANOVAs with Dunnett’s and Tukey’s multiple comparisons post hoc tests).

In this experiment, we pre-treated the neurons with the vehicle, TASE, thymol, and the positive control paclitaxel (a well-known microtubule stabilizer) [20]. On day 2 (DIV 2), the same primary hippocampal neurons days were treated with 50 and 100 nM of nocodazole in the vehicle, as well as TASE (75 µg/mL), thymol (10 µM), and paclitaxel (10 nM). The treatment lasted for 2 h and 24 h continuously. In the 2 h nocodazole treatment group, the culture media was replaced after 2 h of nocodazole treatment with vehicle and treatments containing culture media. Another set of experiments was performed with continuous nocodazole treatment for 24 h. On day 3 (DIV 3), all the control and treatment groups’ neurons were fixed and immunostained with α-tubulin antibody (green) to analyze the neurite area using Neurphology J plug-in (Figure 7a). TASE and thymol significantly (*p* < 0.001) attenuated the nocodazole-induced blunting of microtubule extension in all the treatment groups (Figure 7b). During 2 h and 24 h of nocodazole treatment, thymol treated neurons exhibited a 2-fold increment in the neurite area compared to the vehicle treatment.

### 2.8. TASE and Thymol Promote Dendritic and Axonal Maturation

The degree of arborization of axonal and dendritic trees was studied using Sholl’s analysis [21]. At the point where an axon, dendrite, or branch intersects a particular concentric circle, an axonal or dendritic intersection is noted. We counted the number of branching points that fell within each 10 μm radial circle between two successive concentric circles. Only neurons (>30 cells) that were not intermingled with adjacent neuron processes were chosen for analysis.

#### 2.8.1. TASE and Thymol Promote Dendritic Arborization

Next, we investigated the effects of TASE and thymol on dendritic branching using DIV 5 hippocampal neurons. The cultures were immunostained with α-tubulin (green) and ankyrin G (red, a marker for axon initial segments) and merged to reveal axons (yellow color) (Figure S6a). A significant increment (*p* < 0.05) was observed in the TASE and thymol groups for the number and total length of primary dendrites (~30% and 50–60%, respectively, over vehicle control, *p* < 0.01) (Figure S6b,e). The numbers and lengths of primary and secondary branches were increased in TASE- (~2-fold), thymol-, and stigmasterol-treated (~1.5-fold) neurons as compared to the vehicle control neurons (Figure S6c,f).

Sholl’s analysis of the dendritic arborization indicated an increase in dendritic complexity in TASE- and thymol-treated neurons. The number of dendritic branching points increased by ~2-fold in TASE- (till 90 μm) and thymol-treated neurons (Figure S6d,h), with the total dendritic intersections maximal highest in TASE (~2.8-fold), followed by thymol (~1.7-fold) (Figure S6d). A similar trend was observed for the dendritic intersections, with a ~2-fold increment in TASE- and thymol-treated neurons, which extended up to the circle of 120 μm, whereas the vehicle control neurons extended up to 60 μm (Figure S6g,i). These findings, together with the above results, indicate that TASE and thymol promoted dendritic outgrowth at the early developmental stage, leading to the formation of complex dendritic arborization.

#### 2.8.2. TASE and Thymol Promote Axonal Maturation

Since axons are responsible for the establishment of functional connectivity in neurons, we evaluated whether either TASE or thymol treatments are responsible for axonal growth and maturation using the same neurons analyzed for dendritic arborization.

**Figure 8:**
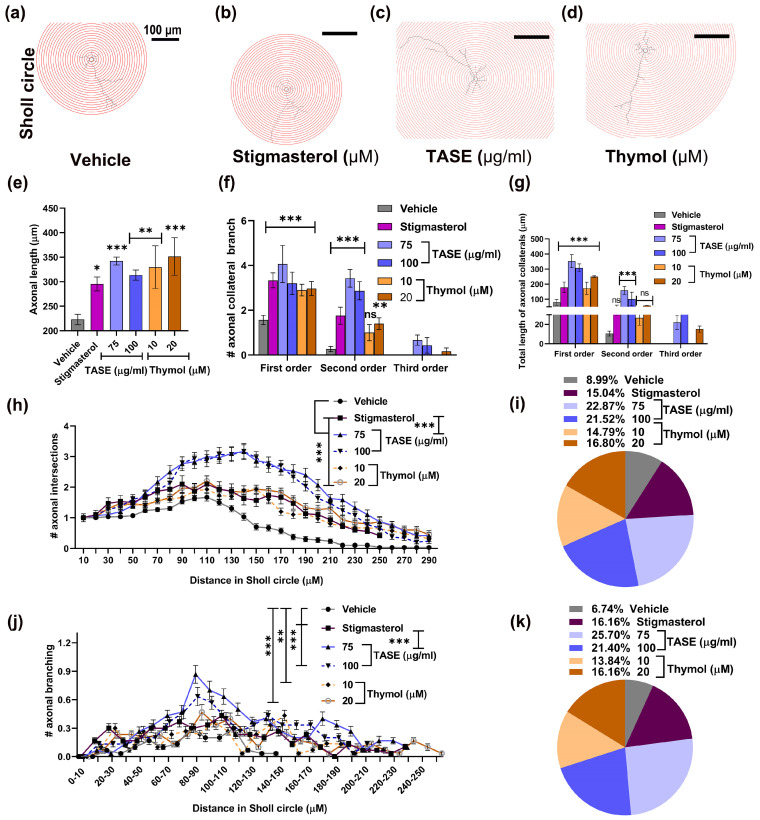
Effects of TASE and thymol on axonal maturation in hippocampal neurons. Neurons were grown under same culture conditions as indicated in Figure 4 for 5 days. Concentric circles of 10 μm extrapolated on neurons for Sholl analysis: (**a**) vehicle, (**b**) stigmasterol, (**c**) TASE, and (**d**) thymol treated neurons. Axonal development assessment: (**e**) length of axonal process. (**f**) Axonal collateral branching number. (**g**) Total length of axon collateral branch according to its order. (**h**) Axonal intersections point in Sholl circle. (**i**) Total axonal intersections in Sholl circle. (**j**) Axon collateral branching points in Sholl circle. (**k**) Total axonal collateral branching points in Sholl circle. Bars and data points represent the mean ± SD (*n* = 30 neurons)**.** Statistical significance compared to vehicle: * *p* < 0.05, ** *p* < 0.01, *** *p*< 0.001, and ns: not significant, one- and two-way ANOVAs with Dunnett’s and Tukey’s multiple comparisons tests.

TASE and thymol significantly increased (*p* < 0.001) the axonal length ~2-fold compared to the vehicle control (Figure 8e). A similar trend was also observed for the number of axon collateral branches (Figure 8f). Moreover, the axonal branching order was increased ~4-fold compared to the vehicle, and third-order axonal branching was observed in the TASE- and thymol-treated neurons (Figure 8g).

Sholl analysis was performed to visualize the axonal maturation in TASE, thymol, and positive control stigmasterol-treated hippocampal neurons. For this purpose, neurons were projected to the concentric circles of 10 μm radius variation (Figure 8a–d). The mean axonal intersections were observed maximal in the TASE- and thymol-treated neurons increased by 2-fold compared vehicle control (Figure 8h) and total intersections were maximal in TASE treatment by ~2.8-fold, followed by the thymol at ~1.8-fold (Figure 8i). Similarly, we observed the axonal collateral branching points, which were extended up to the circle of 260 μm in TASE- and thymol-treated neurons, albeit only up to 150 μm in control neurons (Figure 8j). The total number of axonal collateral branch points were observed maximal in TASE- and thymol-treated neurons with ~ 3-fold and ~2-fold increments, respectively (Figure 8k). Taken together, these findings indicate that TASE and thymol promoted axonal, as well as dendritic, maturation.

## 3. Discussion

In the present study, we reported the neuronal differentiation and outgrowth effects of TASE and its principal component, which is thymol. To investigate in vivo effects, TASE and thymol were supplemented orally to the female mice during the pregnancy and lactation periods, and gene expression in the pups’ brains was analyzed at the mRNA and protein levels for early neuritogenesis markers. TASE and thymol upregulated MAP2 expression, along with the associated early neuritogenic signaling proteins, such as BDNF, NAGK, and NRN1. The neurite outgrowth potential of TASE and thymol in vivo was verified via Golgi–Cox staining in P1 pups’ brains. The in vivo outcomes were validated in vitro through primary hippocampal cultures. The neurons treated with TASE and thymol exhibited well-developed axons and dendrites with higher branching frequency forming a more complex cytoarchitecture. To the best of our knowledge, this is the first study showing the neurotrophin-memetic effects of TASE and thymol, and these findings may be crucial in developing therapeutic approaches to promote brain development and reverse neurodegenerative disorders.

Neurodegenerative disorders, as well as acute brain injuries, are accompanied by extensive damage to the neuronal network. Research indicates that neurotrophin-mimicking substances have the ability to partially repair damaged neural networks via facilitating the regeneration of axons and remodeling of dendrites [22]. This study is the outcome of our continuous effort to identify the natural therapeutic agents reversing the neuronal morphogenesis deterioration in neurodegeneration and other brain injuries. The major components identified in the TASE extracts were thymol (39.1%), p-cymene (30.8%), and γ-terpinene (23.2%) [23]. Among them, thymol is a colorless crystalline monoterpene phenol chemically known as 2-isopropyl-5-methyl phenol, which possesses traditional medicinal values exhibiting various pharmacological properties, including antioxidant, anti-inflammatory, analgesic, antispasmodic, antibacterial, antifungal, antiseptic, and antitumor activities [24]. Similarly, thymol also possesses anticholinesterase, antianxiety, antiepileptic, and antidepressant properties [25,26,27,28].

Pharmacological reports on the neuroprotective potentials of *T. ammi* and its active component thymol directed us to conduct the first report on the neurotrophic study in maternal supplementation and primary hippocampal culture. Thymol is rapidly absorbed in the upper gut after oral administration and degraded in the stomach or intestine [29]. Importantly, thymol is considered safe by the United States Food and Drug Administration due to its negligible toxicity, as evidenced by high LD_50_ values [24,30]. Thymol also has the fascinating ability to cross the blood–brain barrier easily and exert neuroprotection [31]. Thus, we considered the oral maternal supplementation of 50–100 mg/kg of thymol [30] and 250–500 mg/kg of TASE with vehicle and positive control stigmasterol (10 mg/kg) [32] during the pregnancy and the post-natal period. We also verified that these dose ranges exhibited no toxicity with the mice and cells.

Neurons have a distinct polarization that is established in a sequential manner involving five stages, namely neurite sprouting (stage I & II), axonal differentiation (stage III), dendrite arborization (Stage IV), and synaptic formation (stage V) [33]. In this study, the TASE- or thymol-treated neurons at DIV 1 and DIV 2 attained the neuronal polarity comparable to the DIV 3 neurons in the previously reported studies [12,13], where the culture conditions were the same as ours, signifying the prominent neuronal differentiation potential of TASE and thymol. Studies revealed the critical role of maternal diet during the perinatal period in proper neurodevelopment, and its plasticity is vulnerable to nutrition availability [34,35]. Considering the facts, further verification of the in vitro observations was accomplished via the perinatal supplementation of TASE and thymol in female mice.

Neurotrophins are reported to have continuous involvement in enhancing neuronal survival and outgrowth in every stage of development, promoting neuronal activity [4]. Subsequent mRNA and protein level analysis in the pup’s brain exhibited significant upregulation of the markers for early neuritogenesis, such as MAP2, BDNF, NRN1, and NAGK, as compared to the vehicle control. Verification of mRNA changes to the protein translation is crucial to understanding the biological process and cell type involvement. Therefore, immuno-histochemical staining can be the method of choice to assess gene expression at the protein level. Immunostaining also facilitates the quantification and localization of the specific antigen in paraffin-embedded, frozen, or formalin-fixed tissues [36]. For control expressions, we performed immuno-histochemistry with the paraffin-embedded sections of the littermate pups’ brains. The results showed that the expression of BDNF, MAP2, and NAGK was significantly upregulated after the maternal supplementation of TASE or thymol. In addition, Golgi–Cox staining is a valuable method to selectively observe the entire neuronal architecture with clarity, which ultimately facilitates the investigation of the morphological alterations in neurons [37]. Hence, we performed the Golgi–Cox staining in the P1 pup’s brain, and observed a prominent neuronal outgrowth effect mediated using TASE and thymol in prenatal supplementation.

BDNF is secreted at the growth cone during axon–dendrite polarization, which could act as an autocrine factor for axon initiation and growth involving BDNF-induced BDNF release and local insertion of TrkB into the membrane via a positive feedback loop to modulate the neuronal morphology [38]. In this study, ANA-12, which is a TrkB-specific inhibitor, significantly inhibited the TASE- or thymol-mediated neurotrophic activity in primary hippocampal culture, indicating the involvement of BDNF/TrkB signaling-mediated neurotrophic activity. Moreover, the regulation of MAP2 protein is based on the ANA-12 in the TASE and thymol treatments, signifying the role of the TrkB signaling pathway. Dendritic and axonal growth through BDNF/TrkB signaling is mediated through actin and microtubule dynamics [6]. Furthermore, we found that TASE and thymol upregulated BDNF and MAP2 significantly, resulting in the more complex dendritic and axonal arborization in primary hippocampal neurons at DIV 3 and DIV 5 in culture. MAP2 proteins, which are the abundant cytoskeletal components expressed in neurons, may also be involved in initiating nucleation and stabilization of microtubules or microfilaments, maintaining organelle transport within axons and dendrites, and anchoring regulatory proteins such as protein kinases involved in signal transduction [8].

Our finding on the TASE- and thymol-mediated upregulation of BDNF and MAP2 in maternal supplementation in P1 pups is also consistent with the previous phytochemical studies [30,35]. High molecular weight MAP2 was observed in the dendritic growth cones in early neuronal differentiation, suggesting its contribution to neuronal polarization [39]. MAP2 holds the serine- and threonine-rich conserved domain and proline-rich C-terminal domain regulating the protein’s structure and function [40]. Pathogenesis of the MAP2 protein accelerates the disruption of microtubule structure, eventually impairing the axodendritic transport and leading to the apoptosis of neurons [41]. Chen et al. (2012) reported that blocking the microtubule polymerization via nocodazole reduced the number of primary dendrites in hippocampal neurons, which were rescued via BDNF supplementation. In this study, nocodazole blunted microtubule extension in DIV 2 primary hippocampal neurons. This blunting was significantly attenuated through the TASE or thymol treatments, suggesting their roles in microtubule stabilization via BDNF and MAP2 upregulation. However, TASE and thymol treatment could not completely reverse the nocodazole-mediated microtubule disruption. The possible mechanism could be the involvement of partial recovery of some ion channels favoring the nocodazole action. Kalcheva et al. reported that the anchorage of MAP2 increases the stability of microtubules and promotes longer polymerization [42]. In this study, we demonstrated the MAP2 upregulation using TASE and thymol. Therefore, a reasonable hypothesis could be that the more abundant MAP2s led to their more extensive anchorage onto microtubules and resulted in the elongated dendrites and axons in primary hippocampal culture. Thus, TASE and thymol could be possible therapeutic means of promoting neuronal growth impaired in neurodegenerative diseases.

**Figure 9:**
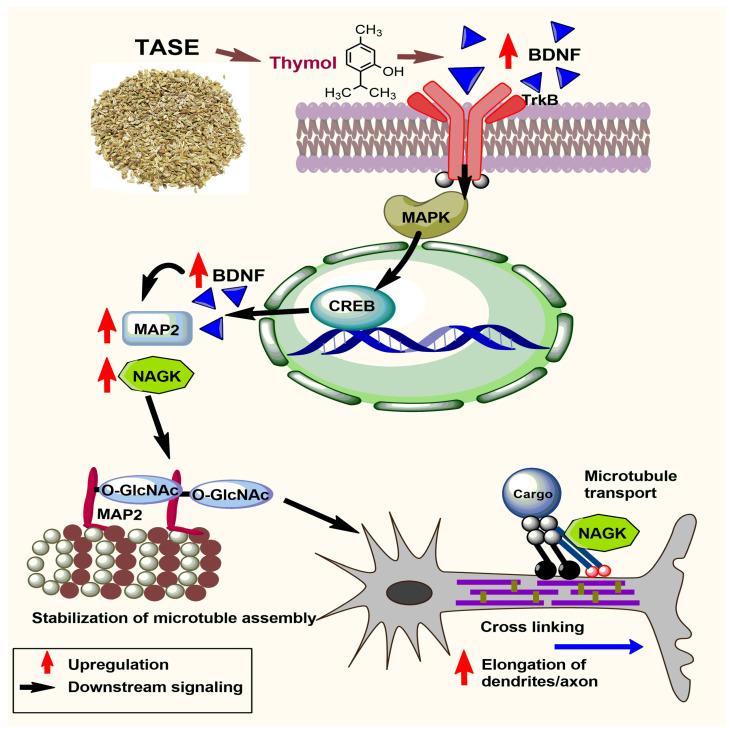
Mechanism of TASE and thymol supplementation in neuronal differentiation and outgrowth effect. TASE and thymol supplementation upregulate the brain-derived neurotrophic factor (BDNF), which follows the TrkB-mediated downstream signaling pathway. Mitogen-activated protein kinase protein (MAPK) is phosphorylated, leading to upregulation of cyclic AMP-responsive element binding protein (CREB) family. CREB family of transcription factors upregulates microtubule-associated protein (MAP2) synthesis. Meanwhile, thymol also upregulates N-acetylglucosamine kinase, which is an enzyme of hexosamine biosynthetic pathway (HBP) which eventually upregulates the synthesis of O-linked β-N-acetylglucosamine (O-GlcNac). Addition of O-GlcNac using O-GlcNAc transferase (OGT) promotes stabilization of MAP2-regulated microtubule assembly. Moreover, MAP2 proteins with modified serine or threonine residues close to phosphorylation sites might be prone to O-GlcNac modification. Cross-linking of microtubules is significantly upregulated, along with NAGK-mediated cargo transport mechanism on the microtubule, finally leading to rapid elongation of dendrites and axons.

We also observed the upregulation of NRN1 using TASE and thymol at the mRNA level in P1 pups. Ogaly et al. reported that thymol treatment increased the expression of BDNF directly through either acting on the BDNF gene or indirect activation of cAMP/CREB signaling, which leads to BDNF expression [30]. The involvement of NRN1 in dendritic outgrowth, maturation, and axonal regeneration via CREB was reported [7]. These previous reports support our finding and, hence, we could assume the involvement of BDNF/TrkB/CREB-mediated gene expression for neurite outgrowth in TASE- and thymol-treated neurons. The present study also showed that TASE and thymol upregulated the NAGK level in P1 pup brains. NAGK is the key building block of N- and O-glycans, generating uridine diphosphate *N*-acetylglucosamine (UDP-GlcNAc) via the hexosamine biosynthetic pathway [43,44]. Reversible modification of the nuclear, cytoplasmic, and mitochondrial proteins is mediated via O-GlcNAcylation and phosphorylation promoting protein association and dissociation, respectively [45,46]. In our study, NAGK was upregulated through TASE and thymol treatments, which might have elevated the association of O-GlcNAc on MAP2 (Figure 9). MAP2 holds the long projection domains extending away from the microtubule, and the addition of carbohydrates may help to maintain extended conformation. Moreover, carbohydrate moieties anchored in the projection domain could also prevent untimely proteolysis of MAP2 [47]. Recently, our laboratory found NAGK-activated dynein, which is a molecular motor complex that transports cargoes along microtubules, thus promoting neurite outgrowth [10,11]. Therefore, TASE and thymol may have elongated the neuronal processes via upregulation of NAGK expression. Based on the present research, we propose a hypothetical signaling pathway for neuritogenesis using thymol, the main component of TASE. 

This study is limited to the TASE and thymol mediated neurite differentiation and outgrowth effect via the BDNF/TrkB signaling pathway. However, the involvement of TASE and thymol in multiple physiological events leading to neuronal differentiation and outgrowth is yet to be studied. Moreover, the variability in the different measures, such as gene or protein expression, as well as neurite outgrowth in TASE and thymol treatments, are dose-dependent and may be associated with the multiple phytochemicals present in TASE. Future studies on other potential phytochemicals in TASE promoting neuronal differentiation and outgrowth are essential to discern the dose-dependent discrepancy.

## 4. Materials and Methods

### 4.1. Chemicals and Reagents

Chemicals and reagents used for the experiments were obtained from Gibco, Thermo Fisher Scientific, Waltham, MA, USA; Sigma Aldrich; and Merk KGaA, Darmstadt, Germany, unless stated otherwise. Thymol (purity > 99%) was purchased from Tokyo Chemical Industry Company Limited (TCI), Tokyo, Japan (Pubchem substance ID: 87572469). Stigmasterol (purity ~95%), nocodazole (purity ~99%), quercetin (purity > 95%) (Pubchem substance ID: 329823865), and paclitaxel (purity > 95%) (Pubchem substance ID: 24277878) were purchased from Sigma-Aldrich, Saint Louis, MO, USA. ANA-12 (Pubchem substance ID: 2799722) was purchased from from Tocris Biosciences, Fischer Scientific, USA.

### 4.2. Plant Sample Collection and Extract Preparation

The seeds of *T. ammi*, which were the product of JK Spices and Food Products (Kolkata, India), were collected from an Asian mart in Cheonan, Republic of Korea. The voucher specimen was deposited in the corresponding author’s laboratory and verified by Dr. Rajendra Gyawali, Associate Professor, Department of Pharmacy, School of Science, Kathmandu University, Dhulikhel, Nepal. The sample was pulverized using a mortar manually. The powdered sample of 2 gm was extracted with 50 mL of 95% (*w*/*v*) ethanol via shaking the mixture at 200 RPM under dark conditions overnight in RT on an orbital shaker (VS-202D, Vision Scientific Co., Ltd., Seoul, Republic of Korea). Thereafter, the mixture was filtered with sterile cotton and centrifuged at 10,000 rpm (17,888× *g*; Sorvall T6000D benchtop centrifuge). The supernatant was concentrated in vacuo and completely dried under a stream of nitrogen gas. The dried TASE extract was weighed, confirming the yield of 3.2% based on weight. An aliquot extract (16 mg/mL) was dissolved in DMSO and stored in the amber vial at −20 °C for further analysis.

### 4.3. Primary Neuronal Culture and Extract/Compound Treatment

In this in vitro experiment, the primary neuronal culture was performed to observe the direct effect of TASE and thymol on neurodevelopment. Pregnant Sprague–Dawley rats were purchased and raised in a light/dark cycle of 12/12 h with access to food and water ad libitum. The procedures for handling animals were conducted according to the standards set forth by Dongguk University’s Institution Animal Care and Use Committee (IACUC-2021-13). On the 19th day of pregnancy, the pregnant rat was euthanized with isofluorane, and the hippocampus of the fetus’ brain was collected in Hank’s balanced salt solution (HBSS) from the random fetus after hypothermia for primary neuronal culture, as previously described [48,49,50]. The hippocampi were dissociated via trypsinization using the graded Pasteur pipettes. The dissociated cells were counted and added to the poly-DL-lysine coated 12 mm glass coverslips. We seeded 10,000 cells/cm^2^ for morphological analysis. The hippocampal cells were seeded in serum-free neurobasal media supplemented with B27, glutamate, and β-mercaptoethanol pre-incubated wells at 37 °C under 5% CO_2_ and 95% air. Extract, compound, or vehicle (DMSO, final concentration <0.5%) was added to the media before the cell seeding. The neuron culture was incubated for 24 h (DIV 1), 2 days (DIV 2), 3 days (DIV 3), or 5 days (DIV 5) for analysis. Live imaging was performed using the Leica DM IL LED microscope to study the neuronal morphology.

### 4.4. In Vivo Mice Experiments

Institute of Cancer Research (ICR) female mice (3 months old) were divided into six groups (*n* = 4 in each group). Animal handling was performed based on the guidelines of the Institution Animal Care and Use Committee of Dongguk University (IACUC-2021-13). The suspension of TASE and thymol, along with the positive control stigmasterol [in a 1% (*v*/*v*) Tween 80 (Sigma-Aldrich, St. Louis, MO, United States), 1% (*v*/*v*) DMSO, 0.9% (*w*/*v*) NaCl solution], was prepared as described previously [17]. Solutions were prepared daily and fed orally from the mating to the post-natal phase. The vehicle control group received 1% (*v*/*v*) Tween 80, 1% (*v*/*v*) DMSO, and 0.9% normal saline (vehicle); the stigmasterol positive control group received stigmasterol (10 mg/kg per day); and the TASE 250 and TASE 500 groups received 250 and 500 mg/kg of extracts orally per day. Mice in the thymol 50 and thymol 100 groups received 50 and 100 mg/kg of thymol orally per day (Figure 1). The appropriate dosing amounts of the TASE, thymol, and stigmasterol were determined based on our previous report [17].

### 4.5. RNA Isolation and Quantitative Real Time-PCR (RT-qPCR) Assay

The whole brain sample (5 mg) was isolated from each pup and washed with chilled PBS thrice. Total RNA was isolated using PureLink™ RNA Mini Kit (12183018A; Thermofischer Scientific), and from the 1 µg of total RNA, cDNA synthesis was carried out using Maxima™ H Minus cDNA Synthesis Master Mix with dsDNase (M1681, Thermofischer Scientific, Waltham, MA, USA), following the manufacturer protocols. Synthesized cDNA was diluted 10 times and 2 µL of diluted cDNA was used in 20 µL of reaction mixture [10 ng of primers (Table S1), 6.5 µL of milli-Q water, 10 µL of PowerTrack™ SYBR Green Master Mix (A46109, Thermofischer Scientific, Waltham, MA, USA) and 0.5 µL of loading dye]. The quantification was performed in StepOne™ Real-Time PCR System (Applied Biosystems, Foster City, CA, USA) using the glyceraldehyde 3-phosphate dehydrogenase (GAPDH) as the endogenous control. Amplification conditions were set up for 2 min at 95 °C, 40 cycles for 15 s at 95 °C, and for 1 min at 60 °C, followed by the melting curve analysis for the specificity of amplification. Finally, relative gene expression was quantified with the normalization of the GAPDH as the endogenous control [51].

### 4.6. Immunocytochemistry

Hippocampal cultures were fixed via a sequential paraformaldehyde/methanol fixation procedure described previously [52]. Immunostaining was performed after blocking the hippocampal cultures, followed by the primary antibody treatment overnight at 4 °C. Primary antibodies used for immunostaining were as follows: primary antibodies to MAP2 (chicken polyclonal clone PA1-10005; 1:5000; Invitrogen, Waltham, MA, USA), tubulin α-subunit (mouse monoclonal 12G10, 1:1000 dilution; Developmental Studies Hybridoma Bank, University of Iowa, Iowa City, IA, USA), ankyrin G (rabbit polyclonal H-215, 1:50 dilution; Santa Cruz Biotechnology Inc., Delaware Ave, CA, USA), tau (rabbit, polyclonal, LF-PA0172, 1:500, Abfrontier, Seoul, South Korea), and Glial fibrillary acidic protein (GFAP) (G-A-5; mouse monoclonal (G3893), 1:200, Sigma Aldrich, Saint Louis, MO, USA). After washing the primary antibody, Alexa-conjugated secondary antibody (Invitrogen) incubation was performed as indicated and mounted on slides. Imaging was performed using the Olympus BX53^®^ polarising light microscope (details in Section 4.10).

### 4.7. Immunohistochemistry

P1 pups were decapitated using cold anesthesia, and brains were isolated and washed in chilled PBS, followed by overnight fixation with 4% paraformaldehyde in PBS (pH 7.4) at 4 °C. The whole brain tissue was washed with PBS twice and dehydrated sequentially in the ethanol gradient xylene, and paraffin embedding was performed. Sagittal sections of 5 μm were prepared using Leica RM 2235 rotary microtome (Leica Biosystems, Nussloch, Germany), and dried overnight at 37 °C, followed by heating for 1 h at 60 °C, and cooled down. After deparaffinization, antigen retrieval was performed through heating the sections with 0.1 M Sodium citrate buffer (pH 6.0) for 15 min at 95°C. Next, the sections were blocked in (1% BSA, 10% goat serum in TBS with 0.025% Triton X-100) for 1 h at RT, followed by the primary antibodies [NAGK (mouse monoclonal G-5, Santa Cruz Biotechnology Inc., Delaware Ave, CA, USA, 1:50)], tubulin [mouse 12G10, 1:50; Developmental Studies Hybridoma Bank, University of Iowa, Iowa City, IA, USA)] and MAP2 [(chicken, polyclonal, PA1-10005; 1:2000), Invitrogen, Waltham, MA, USA] treatment prepared in 1% BSA in TBS with 0.025% Triton X-100 overnight at 4 °C in a humidity chamber. The primary antibodies were washed (3 × 5 min) and incubated with secondary antibodies [Alexa Fluor 488-conjugated goat anti-mouse/rabbit, Alexa Fluor 568-conjugated goat anti-rabbit/chicken at 1:1000 dilutions in 1% BSA in TBS)] for 1 h at RT. The sections were washed with TBS (3 × 5 min) and mounted with Fluoroshield aqueous 4′,6-Diamidino-2-phenylindole dihydrochloride (DAPI) (Abcam, Cambridge, UK) and sealed with the nail polish. Image analysis was performed through obtaining the image intensity ratio with the alpha-tubulin staining in each corresponding field of the image using the Image J intensity measurement tool. IHC imaging was performed via the LEICA confocal laser scanning microscope (TCS SPE); the details are located in Section 4.10.

### 4.8. Golgi-Cox Staining

The pups’ brains were isolated and washed with cold PBS, followed by the brief 4% paraformaldehyde treatment. Golgi–Cox staining was performed through following the standard protocol of the FD rapid Golgistain kit with some modifications (FD Neurotechnologies, Inc., Columbia, MD, USA) [53]. The brains were submerged in solution A/B for 2 weeks, followed by solution C for 72 h. The brains were then embedded in 4% agar gel and sliced at 100 um thickness using a vibratome (DSK microslicer DTK-2000, Ted Pella, Inc., Irvine, CA, USA). The sections were allowed to air dry at room temperature overnight and treated with chilled milli-Q water twice for 4 min. Solution D/E was added for 10 min and washed with milli-Q water twice, and slides were mounted. We imaged the Golgi–Cox staining slides using the Olympus BX53^®^ polarising light microscope (details in Section 4.10).

### 4.9. Western Blotting

The whole brain was washed with ice-cold PBS, homogenized on ice for 30 min using 1 × RIPA buffer containing 1% protease inhibitor, and centrifuged at a speed of 13,000 rpm for 15 min. The supernatant was collected and protein concentrations were determined via the bicinchoninic acid method. The protein sample (40 µg) of each group was separated via SDS-PAGE, transferred to 0.45 μm polyvinylidene difluoride (PVDF) membranes, and blocked in 5% skimmed milk for 1.5 h. The membranes were incubated with primary antibodies overnight at 4 °C, and the goat anti-rabbit/chicken HRP secondary antibodies were incubated for 1 h at room temperature. Primary antibodies used in the western blot analysis were nerve growth factor (NGF) [rabbit, polyclonal, M-20, 1:1000, Santa Cruz biotechnology], brain-derived neurotrophic factor (BDNF) [rabbit, polyclonal, N-20, 1:1000, Santa Cruz Biotechnology, Delaware Ave, CA, USA], MAP2 (chicken, polyclonal, PA1-10005; 1:10,000), Invitrogen], and NAGK [chicken, polyclonal, GW22347, 1:1000, Santa Cruz Biotechnology, and GAPDH [rabbit, monoclonal, 14C10, 1:1000, Cell Signalling technology, Danvers, MA, USA]. The PVDF membranes were soaked in ECL luminous solution and detected using Tanon 5200 Luminescence imaging system. Images were analyzed using the Image J software version 1.43. Each band was compared with the GAPDH band (loading control) in the study.

### 4.10. Microscopic Image Acquisition, Analysis, and Quantification

Phase contrast microscopy images were obtained with a Leica DM IL LED microscope equipped with a Leica DFC3000 G digital monochrome, high-sensitivity, passive-cooled camera (1296 × 966 pixels), which included EXview HAD technology and Leica LAS X software (Leica Microsystems, Wetzlar, Germany) (20X, NA 0.4). Immunocytochemistry and Golgi–Cox images were taken with an Olympus BX53^®^ polarising light microscope assembled with an Olympus DP72^®^ camera at 1920 × 1200 pixels controlled using cellSens™ (Olympus Entry. Ink 2020, Version 1.0) (20X, NA 0.5 and 40X, NA 0.75) (Center Valley, PA, USA). The immuno-histochemistry images were taken using a LEICA confocal laser scanning microscope (TCS SPE) with 63X, NA 1.3 objective lens at 405 nm and 532 nm (Leica Microsystems, Wetzlar, Germany). The representative images were processed using Adobe Photoshop 7.0 software (San Jose, CA, USA). Image analyses for the morphometry and quantitative analysis were performed, as described previously [13], with Image J software (version 1.49) and using the simple neurite tracer (National Institute of Health, Bethesda, MD, USA), concentric circle, and Sholl plug-ins (http://biology.ucsd.edu/labs/ghosh/software (accessed 14 January 2023)). The neurite area for the nocodazole-treated and control neurons was evaluated using the Neurphology J plug-in [54] with Image J software (version 1.43).

### 4.11. Statistical Analysis

The results were expressed as the mean ± SD with at least three independent experiments unless stated otherwise. Statistical analysis was performed using the GraphPad Prism version 8.0.0 for Windows (San Diego, CA, USA). The statistical difference was obtained using Student’s *t*-test, one-way and two-way ANOVA, and Dunnett’s and Tukey’s multiple comparisons tests; statistical significance was determined at *p* values ≤ 0.05.

## 5. Conclusions

In conclusion, our study emphasizes the neuronal differentiation and neuritogenic activity of TASE and its principal component thymol in both pre-natal maternal supplementation and primary hippocampal neurons. TASE and thymol upregulated BDNF/TrkB signaling and promoted the synthesis of MAP2 proteins, which stabilized and elongated the microtubule and resulted in early neuronal differentiation, dendritic arborization, and axonal maturation modulating activities. TASE and thymol might have clinical significance for the treatment of various neurodegenerative disorders and acute brain injuries. Thus, our findings are helpful in developing natural therapeutic agents to prevent neurodegeneration and related disorders.

## Data Availability

The data that support the findings of this study are available from the corresponding author upon reasonable request.

## Supplementary Materials

The supporting information can be downloaded at: https://www.mdpi.com/article/10.3390/ijms24108565/s1. References [55,56,57,58,59,60,61] are cited in the Supplementary Materials.
