# Supplementary material for: Neuronal Differentiation and Outgrowth Effect of Thymol in Trachyspermum ammi Seed Extract via BDNF/TrkB Signaling Pathway in Prenatal Maternal Supplementation and Primary Hippocampal Culture
Source: Int J Mol Sci. 2023 May 10;24(10):8565. doi: 10.3390/ijms24108565 (PMC10218397; doi:10.3390/ijms24108565)
Supplement: Supplementary file 1 [file ijms-24-08565-s001.zip › ijms-2381397-supplementary.pdf]

Supplementary File

Article

Neuronal differentiation and outgrowth effect of thymol in *Trachyspermum ammi* seed extract via BDNF/TrkB signaling pathway in prenatal maternal supplementation and primary hippocampal culture

Binod Timalsina [1], Md Nazmul Haque [1,2], Raju Dash [1], Ho Jin Choi [1], Nisha Ghimire [3] and Il Soo Moon [1,*]

[1] Department of Anatomy, Dongguk University College of Medicine, Gyeongju 38066, Republic of Korea
[2] Department of Fisheries Biology and Genetics, Patuakhali Science and Technology University, Patuakhali 8602, Bangladesh
[3] Department of Life Science and Biochemical Engineering, Graduate School, SunMoon University, Asan 31460, Republic of Korea
* Correspondence: moonis@dongguk.ac.kr

Supplementary Methods

S1. Ultra-High Performance Liquid chromatography (U-HPLC)

The active compounds in the *Trachyspermum ammi* seed extract (TASE) were quantified using ultra-high performance liquid chromatography (U-HPLC) [1]. For sample preparation, thyme extract and thymol standard were dissolved in HPLC-grade methanol and filtered with a 0.2-μm Whatman filter. Furthermore, the sample was injected (20 ul) in U-HPLC, which was purchased from Thermo Fischer Scientific, Massachusetts, USA, separated using a Mightysil reverse-phase C18 column (4.6 × 250, 5 μm; Kanto Chemical, Tokyo, Japan), and analyzed via photodiode array (PDA) detector with absorbance spectrum set at 270 nm. For mobile phases, water (A) and acetonitrile (B) were used in a gradient system of B at 10% for 0–1min, 50% for 1–8 min, 70% for 8–14 min, 95% for 14–16 min, and 10% for 16-25 min, all at a flow rate of 1 ml/min. A Thymol standard curve was prepared, and the amount of thymol was quantified as 33.7% of the extract.

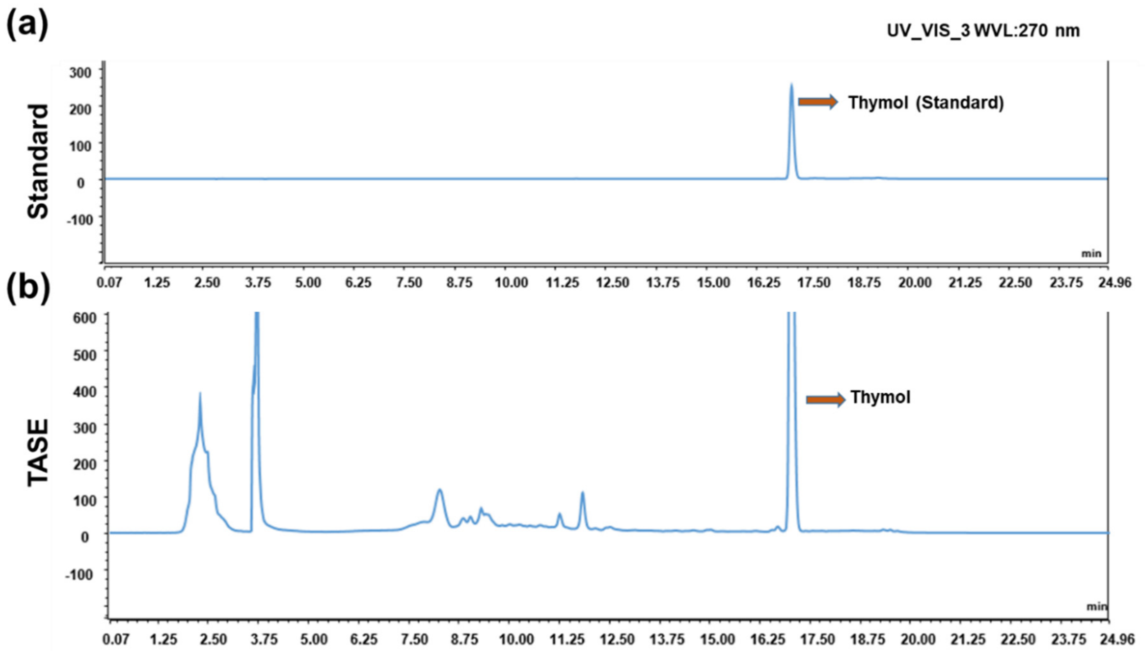

Figure S1. U-HPLC analysis of *Trachyspermum ammi* seed extract (TASE). (**a**) Thymol standard peak was observed at a retention time of 17.106 minutes. (**b**) TASE dissolved in methanol was injected for U-HPLC, and major active phenolic constituent peaks were observed with thymol compound as major peak corresponding to thymol standard peak.

S2. Identification of glia percentage in primary hippocampal culture

The primary hippocampal culture was maintained until DIV 3 and DIV 14 in the routine neuronal culture medium used for the analysis in this study.

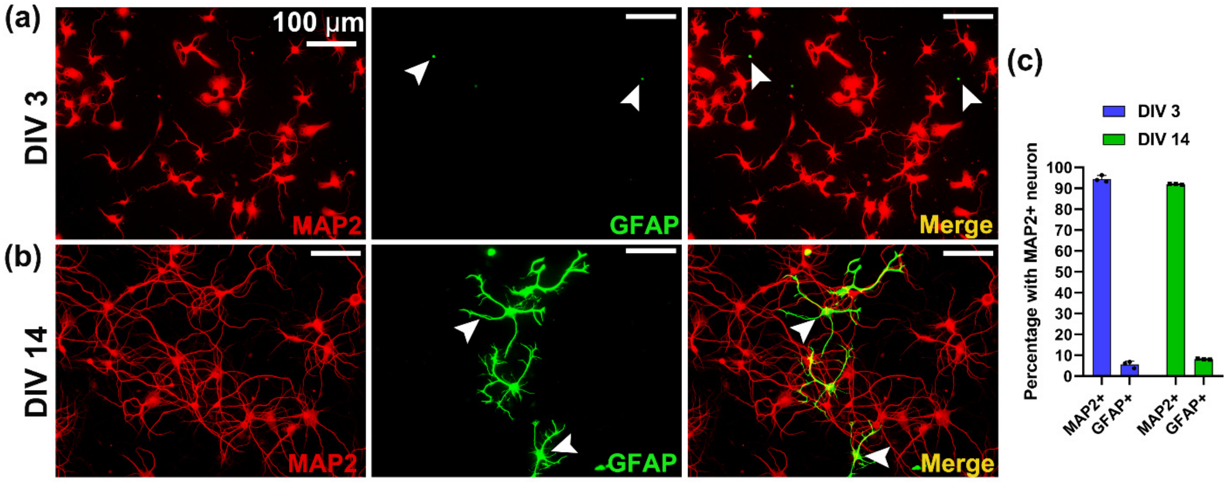

Figure S2. Immuno-cytochemistry showing the neuron- and glia-specific staining. Neuron- (MAP2, red) and glia-specific (GFAP, green) markers were used and pointed out using arrowheads. (**a**) and (**b**) representative images at DIV 3 and DIV 14. (**c**) percentage of MAP2- and GFAP-positive cells as percentages are shown in DIV 3 and DIV 14. MAP2- and GFAP-positive cells percentages were calculated through counting total cells.

S3. RT-qPCR analysis of NRN1 gene for P1 pups brain lysate

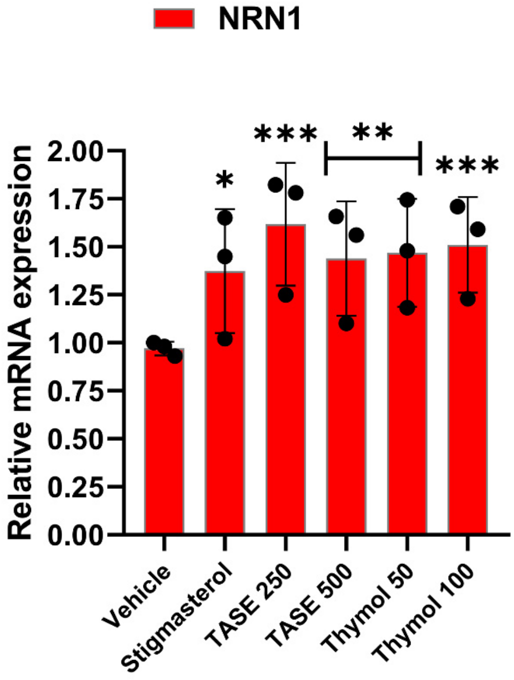

Figure S3. RT-qPCR analysis of *NRN1* gene in P1 pups whole brain lysate. One-way ANOVA with Dunnett's and multiple comparisons was used for statistical analysis. * $p < 0.05$, ** $p < 0.01$, and *** $p < 0.001$ represent difference between vehicle control and treatments groups. ($n = 3$ pups from different mothers in same treatment group).

S4. Effect of TASE and thymol on BDNF expression in the mouse pup's brains

The experimental mice were continuously fed with the vehicle, TASE, and thymol in the lactation stage. At post-natal day 12 (P12), the mouse pups' brains were collected from different groups, and the level of mature BDNF was observed via western blot assay. BDNF expression was significantly upregulated in the pup's brain via the TASE 500 mg/kg ($p < 0.01$) and thymol 50 mg/kg ($p < 0.01$) supplementation in the lactating mothers.

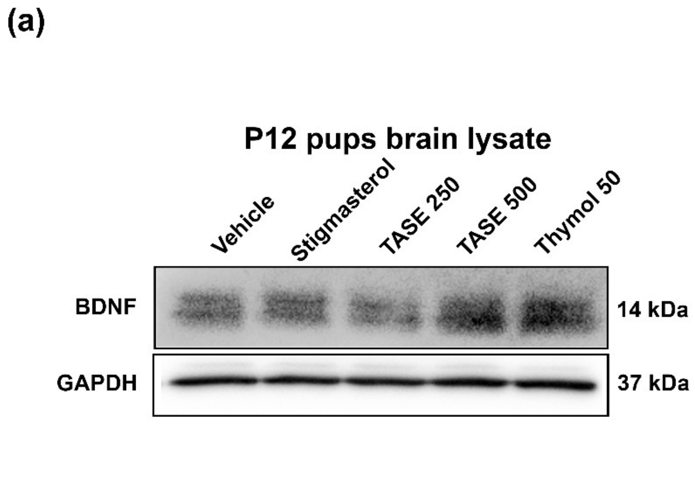

Figure S4. Western blot analysis of mouse pups' brains lysate at P12 stage. (**a**) Western blot band analysis showing BDNF expression in mouse pups' brains at P12. (**b**) Relative BDNF expression of TASE and thymol treatments with loading control (GAPDH). One-way ANOVA with Dunnett's and multiple comparisons was used for statistical analysis. ** $p < 0.01$, and *** $p < 0.001$ represent difference between vehicle control and treatments groups. ($n = 3$ pups from different mothers in same treatment group).

S5. Analysis of Neuronal Viability

Neuronal cultures were incubated with either vehicle or TASE and thymol with variable concentrations for 8 days. Viable cells were counted via trypan blue exclusion assay following the procedure described previously [2, 3]. The cells were washed with Dulbecco's phosphate buffered saline (D-PBS, Invitrogen) and stained with 0.4% trypan blue for 15 min at 37°C. After washing the trypan blue dye, the cells were fixed with 4% paraformaldehyde. Since this assay is based on the membrane integrity of the cell, dead neurons are stained with dark-blue color due to the permeable membrane, whereas live neurons have intact membrane integrity, preventing the permeability of dye. Cells on two coverslips, with 400-500 cells per coverslip, were counted randomly. Viability was determined as the percentage of the ratio of the number of unstained cells (live neurons) to the total number of cells counted (live plus dead neurons), as shown Figure S4.

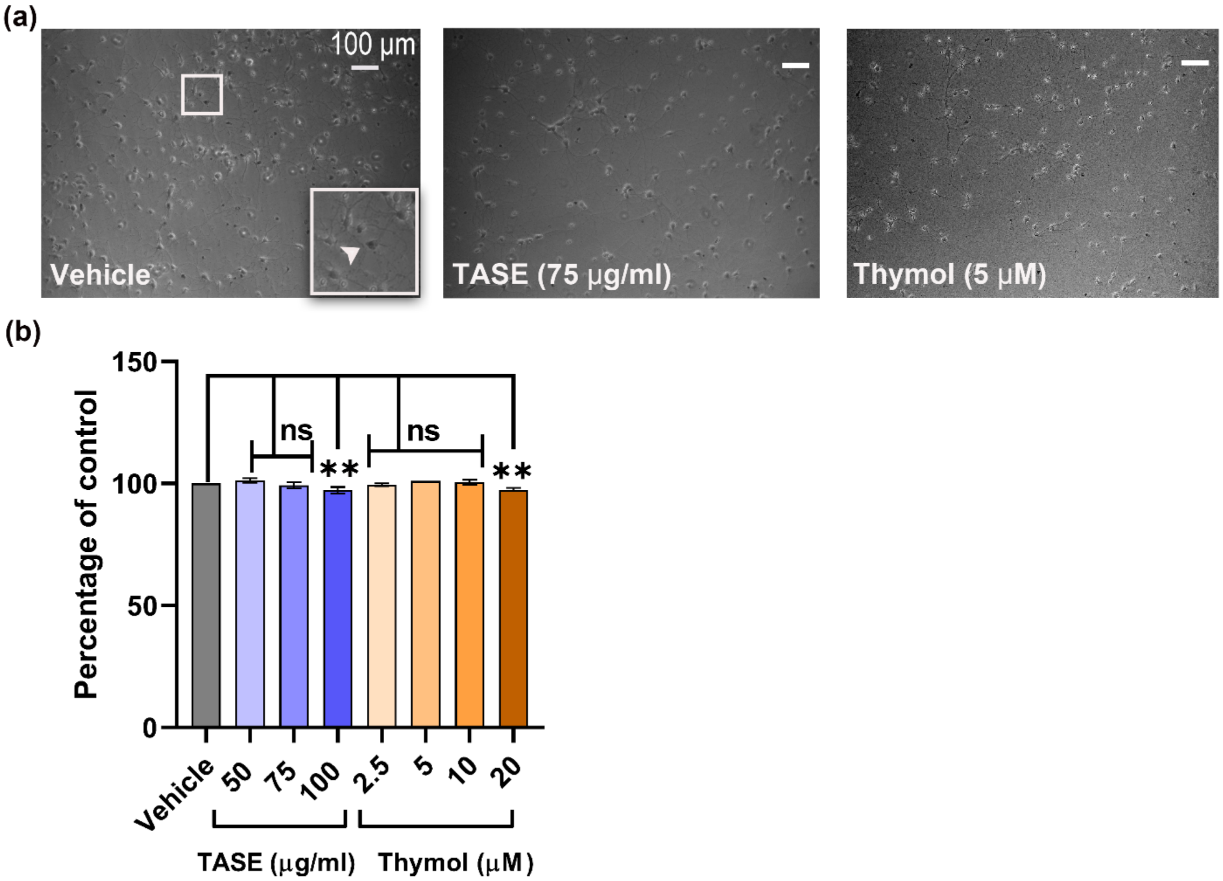

Figure S5. Trypan blue exclusion assay for neuronal viability with treatments TASE and thymol. Rat hippocampal neurons were cultured with vehicle, TASE, and thymol for 8 days. Neuronal viability was assessed via trypan blue exclusion assay. (**a**) Images for trypan blue assay for vehicle, TASE, and thymol treatments, respectively. Dead neuron is stained dark-blue, as indicated with arrowhead. Scale bar, 100 μm, applied to all images. (**b**) Neuronal viability is determined as proportion of unstained cells (live neurons) to total number of cells counted (live plus dead neurons); viability of control culture is normalized to 100%. Bars represent mean ± SD ($n = 4$, each with a minimum of 500 neurons). ** $p < 0.01$ with one-way ANOVA (Dunnett's multiple comparisons post hoc test) represent difference between vehicle control and treatment groups.

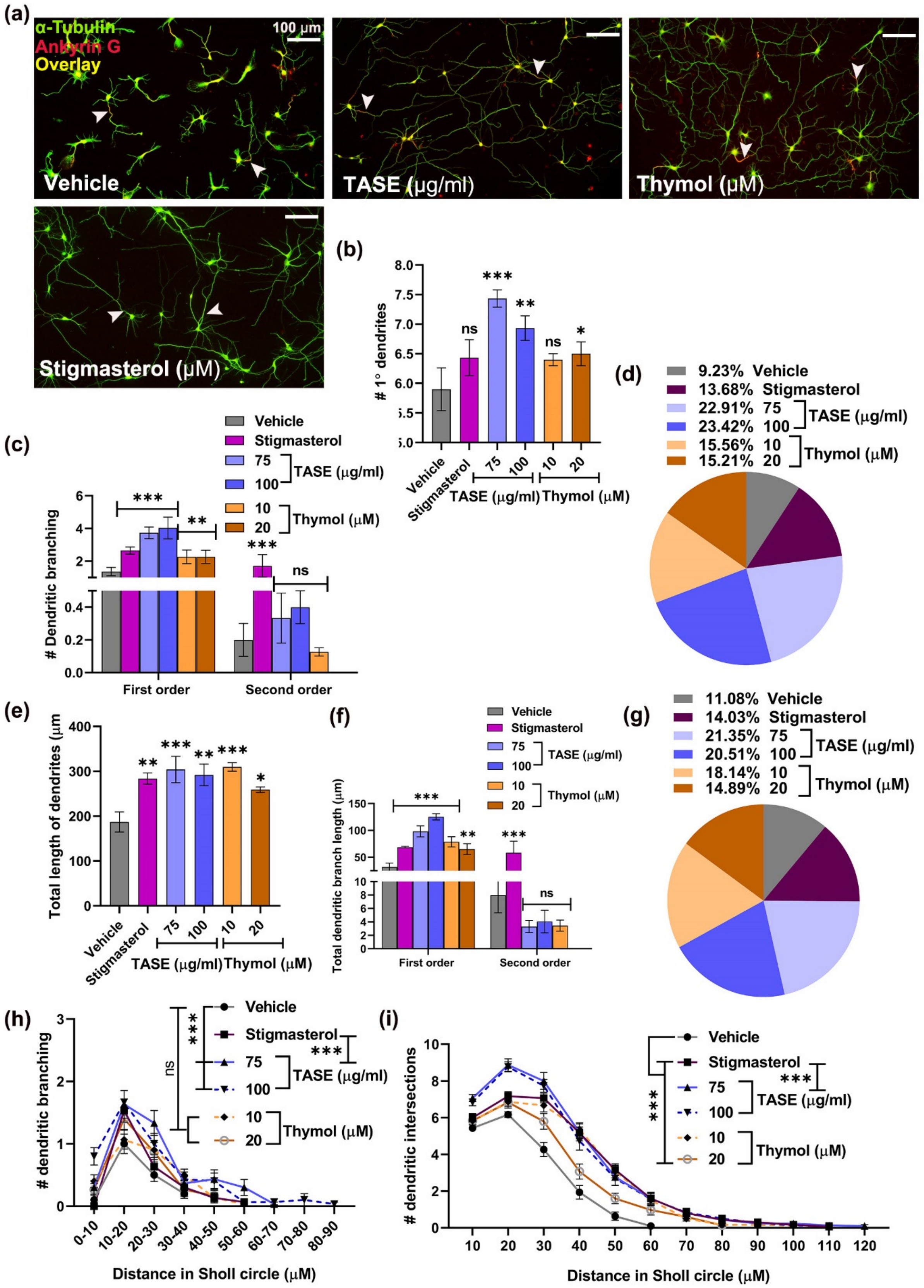

Figure S6. TASE and thymol promote dendritic arborization in hippocampal neurons. Hippocampal cultures were maintained for 5 days on same culture conditions. Immunostaining was performed using α-tubulin (green) and ankyrin G (red) primary antibodies. Axons are pointed out with arrowheads to show the Ankyrin G concentrated area at axon initial segment. (**a**) Immunofluorescence images are represented from hippocampal cultures at DIV 5 to visualize morphological dif-

ferences with vehicle and stigmasterol as control. Scale bar, 100 µm, applies to all images. (**b**) Number of primary dendrites at DIV 5 neurons. (**c**) Number of dendrites branching. (**d**) Total dendritic branching points in Sholl circle. (**e**) Length of dendrites. (**f**) Total length of dendritic branching order. (**g**) Total dendritic collateral branch points intersections in Sholl circle. (**h**) Sholl analysis for dendritic branching and (**i**) dendritic intersections points in DIV5 hippocampal neurons. Data points represent mean ± SD ($n = 30$ neurons from three individual experiments). Statistical significance was compared to vehicle using one- and two-way ANOVAs with Dunnett's and Tukey's multiple comparisons tests: * $p < 0.05$, ** $p < 0.01$, and *** $p < 0.001$.

Table S1. Primers used for amplification of neuritogenesis marker genes in RT-qPCR analysis.

<table>
<tr><th>Genes</th><th>Forward (5′-3′)</th><th>Reverse (5′-3′)</th><th>References</th></tr>
<tr><td>NGF</td><td>GCATGGCCAACTACC C</td><td>CCTTCCTGGCAATCCGT</td><td>[4]</td></tr>
<tr><td>BDNF</td><td>TGCAGGGGCATAGACAAAAGG</td><td>CTTATGAATCGCCAGCCAATTCTC</td><td>[5]</td></tr>
<tr><td>NAGK</td><td>GTGCTCATATCTGGAACAGG</td><td>ACCCTCATCACCCATCATA</td><td>[6]</td></tr>
<tr><td>MAP2</td><td>TCTAAAGAACATCCGTCACAGG</td><td>GGTGAGCATTGTCAAGTGAGC</td><td>[7]</td></tr>
<tr><td>NRN1</td><td>GCATGGCCAACTACCC</td><td>CCTTCCTGGCAATCCGT</td><td rowspan="2">[8]</td></tr>
<tr><td>GAPDH</td><td>TGACGTGCCGCCTGGAGAAA</td><td>AGTGTAGCCCAAGATGCCCTTCAG</td></tr>
</table>

References

1. Hajimehdipoor, H.; Shekarchi, M.; Khanavi, M.; Adib, N.; Amri, M., A validated high performance liquid chromatography method for the analysis of thymol and carvacrol in Thymus vulgaris L. volatile oil. *Pharmacogn Mag* **2010**, *6*, 154-158.
2. Strober, W., Trypan blue exclusion test of cell viability. *Current protocols in immunology* **2001,** Appendix 3, Appendix 3B.
3. Hannan, M.A.; Kang, J.Y.; Mohibbullah, M.; Hong, Y.K.; Lee, H.; Choi, J.S.; Choi, I.S.; Moon, I.S., Moringa oleifera with promising neuronal survival and neurite outgrowth promoting potentials. *Journal of ethnopharmacology* **2014**, *152*, 142-50.
4. Yuan, H.; Du, S.; Chen, L.; Xu, X.; Wang, Y.; Ji, F., Hypomethylation of nerve growth factor (NGF) promotes binding of C/EBPα and contributes to inflammatory hyperalgesia in rats. *Journal of Neuroinflammation* **2020**, *17*, 1-11.
5. Huang, T.; Krimm, R.F., Developmental expression of Bdnf, Ntf4/5, and TrkB in the mouse peripheral taste system. *Developmental Dynamics* **2010**, *239*, 2637-2646.
6. Campbell, S.; Mesaros, C.; Izzo, L.; Affronti, H.; Noji, M.; Schaffer, B.E.; Tsang, T.; Sun, K.; Trefely, S.; Kruijning, S., Glutamine deprivation triggers NAGK-dependent hexosamine salvage. *Elife* **2021**, *10*, e62644.
7. Bora, G.; Sucularli, C.; Hensel, N.; Claus, P.; Yurter, H.E., Investigations of microtubule-associated protein 2 gene expression in spinal muscular atrophy. **2019**.
8. Wan, K.; Mao, F.; Li, Q.; Wang, L.; Wei, Z.; Wang, P.; Liao, X.; Xu, M.; Huang, J.; Pan, Z., Neuritin-overexpressing transgenic mice demonstrate enhanced neuroregeneration capacity and improved spatial learning and memory recovery after ischemia-reperfusion injury. *Aging* **2021**, *13*, 2681.
